# Quality of life and musculoskeletal symptoms of nurses in Primary Care in southern Brazil

**DOI:** 10.1590/0034-7167-2023-0342

**Published:** 2025-03-14

**Authors:** Jadieli Simoni Roll-Koch, Lirane Elize Defante Ferreto, Adilson Carlos da Rocha, Rosebel Trindade Cunha Prates, Dalila Moter Benvegnú, Gisele Arruda, Ana Paula Vieira, Franciele Aní Caovilla Follador

**Affiliations:** I Universidade Estadual do Oeste do Paraná. Francisco Beltrão, Paraná, Brazil; II Universidade Federal da Fronteira Sul. Realeza, Paraná, Brazil

**Keywords:** Quality of Life, Musculoskeletal Pain, Nurses, Primary Health Care, Occupational Health, Calidad de Vida, Dolor Musculoesquelético, Enfermeras y Enfermeros, Atención Primaria de Salud, Salud Laboral

## Abstract

**Objectives:**

to assess knowledge about quality of life and musculoskeletal symptoms of Primary Healthcare nurses in the Francisco Beltrão Region, Paraná.

**Methods:**

an exploratory, quantitative, cross-sectional study carried out with nurses, using a sociodemographic questionnaire, WHOQOL-BREF and the Nordic Musculoskeletal Symptoms Questionnaire. Statistical analysis used the Statistical Package for the Social Sciences statistical program, applying chi-square test, Kruskal-Wallis test and Mann-Whitney U test.

**Results:**

the sample consisted of 66 nurses, the majority of whom were women (86.4%), married (71.2%) and had a mean age of 37.23 years. When assessing QoL, the physical domain obtained the lowest average (55.74), followed by the psychological domain (60.67). Regarding musculoskeletal symptoms, pain in the lower back was the most cited (75.80%).

**Conclusions:**

the knowledge that nurses have about quality of life shows that it is influenced by muscle pain, having another employment relationship and the use of psychotropic drugs.

## RESUMEN

**Objetivos:**

evaluar el conocimiento sobre calidad de vida y síntomas musculoesqueléticos de enfermeros que actúan en Atención Primaria de Salud en la Región Francisco Beltrão, Paraná.

**Métodos:**

se trata de una investigación exploratoria, cuantitativa, transversal, realizada con enfermeras, utilizando un cuestionario sociodemográfico, WHOQOL-BREF y Cuestionario Nórdico de Síntomas Musculoesqueléticos. El análisis estadístico se utilizó mediante el programa estadístico *Statistical Package for the Social Sciences*, aplicando la prueba de chi cuadrado, la prueba de Kruskal-Wallis y la prueba U de Mann-Whitney.

**Resultados:**

la muestra estuvo compuesta por 66 enfermeros, la mayoría mujeres (86,4%), casadas (71,2%) y edad promedio de 37,23 años. Al evaluar la CV, el dominio físico obtuvo el promedio más bajo (55,74), seguido del dominio psicológico (60,67). En cuanto a los síntomas musculoesqueléticos, el dolor en la zona lumbar fue el más citado (75,80%).

**Conclusiones:**

el conocimiento que tienen los enfermeros sobre la calidad de vida muestra que está influenciado por el dolor muscular, tener otro trabajo y el uso de psicofármacos.

## INTRODUCTION

Primary Healthcare (PHC) is responsible for serving the largest population contingent, with different health demands. These professionals work in the prevention, promotion and recovery of the population’s health, often with few resources and high demands. Nurses are among the professionals on the team that make up primary care line. Generally, this professional is responsible for population management, organization and care, i.e., numerous activities to be carried out during working hours^(1)^.

PHC is of essential importance, as it aims to provide comprehensive care to individuals, serving as their gateway to healthcare services^(2)^. In addition to this comprehensive care, it consists of strategies for better provision of this service, such as the Family Health Strategy (FHS) and the Family Health Unit (FHU)^(3)^.

The Ministry of Health (MoH) itself reinforces the importance of care provided by multidisciplinary teams, and in these teams, the importance of the role played by nurses as managers and providers of care in prevention and health promotion actions is evident. However, many times, dedication to improving the health of the population they serve is not seen in themselves^(1)^. When personal care is not a priority in their lives, there is a decline in the quality of life (QoL) of these professionals^(4)^.

QoL, according to the World Health Organization (WHO), is “[...] individuals’ perceptions of their position in life in the context of the culture and value systems in which they live and in relation to their goals, expectations, standards and concerns”^(5)^. Therefore, it is influenced by external and internal factors, such as pain and discomfort, social relationships, the course of thought, physical safety and leisure^(6)^ .

Nurses have prior knowledge about QoL, however, most of them are dissatisfied and report having difficulty maintaining a balance between personal and professional life^(1)^. They also know the importance of having time dedicated to leisure activities, which are not linked to the work environment and process, however, the lack of time does not favor performing these activities, interfering with personal life and even in the care provided to patients^(4)^. Currently, we can mention that the COVID-19 pandemic has influenced the lack of social interaction and the performance of activities outside the home. With the uncontrolled spread of the virus and the lack of a cure and immunobiological for prevention, the entire population experienced quarantine^(6)^.

With continued exposure to stressors and long working hours, these professionals consequently began to experience muscle pain caused by tension, poor posture and the repetitiveness of an action without adequate ergonomics^(7)^. Work-related musculoskeletal disorders (WMSDs) are the result of untreated and unprevented repetitive strain injuries (RSIs). They are considered the main causes of absenteeism and presenteeism at work, in addition to being the result of professional shortages and environments that are not ergonomically adapted^(8)^. A study conducted in Vietnam also stated that female professionals are more predisposed to developing RSIs and WMSDs due to double shifts, such as household chores^(9)^. Consequently, this symptomatology results in failures in patient care, self-medication and also a decrease in QoL^(10)^. With this problem, in which nurses perform diverse functions in their work environment, as well as the workday, it is necessary to understand how this professional’s QoL is, as well as the symptoms resulting from work and the frequency of pain, seeking knowledge so that the class and the system seek improvements to minimize such occurrences.

## OBJECTIVES

To assess knowledge about QoL and musculoskeletal symptoms of PHC nurses of the Francisco Beltrão Region, Paraná.

## METHODS

### Ethical aspects

The study was approved by the *Universidade Estadual do Oeste do Paraná* (UNIOESTE) Research Ethics Committee (REC), and data were only collected after signing the Informed Consent Form.

### Design, study location and period

This is an exploratory, quantitative, cross-sectional study conducted with nursing professionals working in the PHC of the Francisco Beltrão Health Region, in Paraná. This health region includes a total of 27 municipalities and is part of the Western Paraná macro-region^(11)^. In this health region, PHC consists of 213 nurses, according to data obtained from the Brazilian National Registry of Health Establishments (CNES - *Cadastro Nacional de Estabelecimentos de Saúde*) in March 2021^(12)^. Data collection was carried out between May 2021 and March 2022, during the COVID-19 pandemic. Moreover, the STrengthening the Reporting of Observational studies in Epidemiology (STROBE) guidelines for publishing observational studies were followed^(13)^.

### Sample, inclusion and exclusion criteria

The sample was constituted by convenience/acceptance. Initially, the data collection method was presented online, aided by Google Forms^®^. The link containing the questionnaires was sent to all nurses via email and/or WhatsApp^®^. After low adherence, collection was carried out in person in all municipalities. Therefore, the sample consisted of 66 nurses working in PHC.

### Study protocol

For this study, sociodemographic and epidemiological questionnaires were used as data collection instruments, which addressed data such as age, sex, marital status, length of service, monthly family income, whether workers have a graduate degree, whether they engage in physical activity, religion, other employment relationships, number of hours worked per week, whether the workers work at night, underlying diseases, medications used for mental health and whether the workers started using these medications after the start of the pandemic.

In order to assess nursing professionals’ QoL, the WHOQOL-BREF questionnaire was used, which is an abbreviation of WHOQOL-100^(14)^. In 2000, this questionnaire was translated, applied in the Portuguese version and validated by the Brazilian literature^(15)^. It consists of 26 questions, two of which refer to QoL in general and health status, and the remaining 24 questions assess four domains separately, namely: physical; psychological; social relations; and environment^(14)^.

To assess musculoskeletal symptoms, the Nordic Musculoskeletal Symptoms Questionnaire (NMSQ) was used. In this study, the version translated and validated for Portuguese by Mesquita (2010)^(16)^ was used. This questionnaire assesses whether a respondent has presented symptoms of pain, discomfort or numbness in their respective body segments in the last 12 months and in the seven days prior to completing the form. It also asks whether this employee had to take time off or avoid their normal activities due to musculoskeletal problems in the segments^(17)^.

The literature has already demonstrated that the questionnaires used, although in the Portuguese version, presented similar results regarding psychometric characteristics^(15)^. In this regard, their use is justified due to instrument availability and the similarity between semantics of languages, and during the research, no reports were observed from participants about any interpretation problems due to their use. Thus, no harm was observed in the use of the Portuguese version.

### Analysis of results, and statistics

The questionnaires adopted and applied to nurses were coded with the aid of Microsoft Excel^®^, and, for statistical analyses and associations, Statistical Package for the Social Sciences (SPSS) version 23 was used, in which non-parametric tests were applied, such as the chi-square test, Kruskal-Wallis test and Mann-Whitney U test, with correction for sample.

## RESULTS

The sample consisted of 66 nursing professionals, the majority (86.4%) of whom were female, with a mean age of 37.23, with a minimum of 23 and a maximum of 56 years. Of these, 71.2% were married, white (94.4%), Catholic (81.1%), with one child (37.9%) and a family income of more than four minimum wages (60.6%).

As for the time since graduation of these professionals, the majority had graduated more than ten years ago (59.1%) and had graduate degrees (84.8%). It was evident that 53% had been working in the area for more than ten years; few of them had another employment relationship (12.1%); and they worked night shifts (12.1%). In addition to this, the majority (86.4%) worked 30 to 44 hours per week.

Periodic leisure activities were reported by 83.3% of participants. Regular physical activity was reported by only 42.4% of them. When asked about alcohol use, 57.6% reported using it at least socially, and 93.9% reported not being smokers. Some of these professionals (24.2%) reported having underlying diseases, including hypertension (HT), diabetes mellitus (DM), asthma, heart disease, hypothyroidism, psoriasis, chronic obstructive pulmonary disease (COPD), obesity, essential action tremor, RSI and WMSD.

The presence of mental illness was reported by 22.7%, however, of all interviewees, 28.8% reported the use of psychotropic medications, and only 15.2% stated that they had undergone psychotherapy. When asked about whether they had started using psychotropic medications after the beginning of the pandemic, only 7.6% said so, and 15.2% were already using the medication and increased the dose during the pandemic. When asked if there had been a change in QoL after experiencing the pandemic, 75.8% said yes, but it had worsened.

Muscle pain is a discomfort that can affect the entire population. To identify the prevalence of musculoskeletal symptoms among nurses, the QNSM was adopted, which obtained the results described in Tables 1 and 2.

**Table 1:** Musculoskeletal symptoms described by nurses from the Francisco Beltrão Health Region, Paraná, Brazil

Location/region of pain	Pain last 12 months		Impediment to carrying out activities in the last 12 months		Pain in the last seven days	
	n	%	n	%	n	%
Neck	45	68.2	18	27.3	24	36.4
Shoulders both	28	42.4	5	7.6	9	13.6
Right shoulder	8	12.1	7	10.6	6	6.1
Elbow both	2	3.0	2	3.0	2	3.0
Right elbow	7	10.6	3	4.5	2	3.0
Left elbow	1	1.5	1	1.5	-	-
Wrists and hands	8	12.1	2	3.0	1	1.5
Right wrist and hand	9	13.6	5	7.6	3	4.5
Left wrist and hand	3	4.5	1	1.5	1	1.5
Thoracic region	20	30.3	7	10.6	10	15.2
Lower region	50	75.8	22	33.3	26	39.4
Hips and thighs	8	12.1	3	4.5	5	7.6
Knees	20	30.3	7	10.6	10	15.2
Ankle and feet	11	16.7	2	3.0	5	7.6

**Table 2:** Association of musculoskeletal symptoms by anatomical region in relation to the sex of nurses in the Francisco Beltrão Health Region, Paraná, Brazil

			Sex				*p* value
			Female		Male		
			n	%	n	%	
Neck	12m		42	66.64	3	4.54	0.047^*^
	7d		22	33.33	2	3.03	0.476
	Impediment		17	25.76	1	1.52	0.317
Shoulders	12m		32	48.48	4	6.06	0.954
	7d		12	18.18	2	3.03	0.667
	Impediment		10	15.15	2	3.03	0.284
Elbows	12m		8	12.12	2	3.03	0.054
	7d		4	6.06	0	0.00	0.746
	Impediment		5	7.58	1	1.52	0.047^*^
Fists and hands	12m		18	27.27	2	3.03	0.928
	7d		4	6.06	1	1.52	0.667
	Impediment		8	12.12	0	0.00	0.740
Thoracic	12m		18	27.27	2	3.03	0.728
	7d		10	15.15	0	0.00	0.202
	Impediment		6	9.09	1	1.52	0.853
Lumbar	12m		44	66.67	6	9.09	0.957
	7d		24	36.36	2	3.03	0.374
	Impediment		21	31.82	1	1.52	0.182
Hips and thighs	12m		8	12.12	0	0.00	0.262
	7d		5	7.58	0	0.00	0.388
	Impediment		3	4.54	0	0.00	0.510
Knees	12m		16	24.24	4	6.06	0.823
	7d		9	13.63	1	1.52	0.196
	Impediment		4	6.06	3	4.54	0.080
Ankles and feet	12m		9	13.64	2	3.03	0.500
	7d		4	6.06	1	1.52	0.574
	Impediment		1	1.52	1	1.52	0.096

* *Significant difference at 5% level (p<0.05).*

The data presented in Table 1 refer to musculoskeletal symptoms reported by nurses. When asked if they had any problems such as pain, discomfort or numbness in the last 12 months, the lumbar region was the most cited (75.80%); considering the last seven days, it was cited in 39.4%. When asked if pain prevented them from carrying out normal activities, such as work, housework or hobbies, in the last 12 months, it was cited in 33.30%. Subsequently, in relation to position as pain, discomfort or numbness, in the last 12 months, the neck region was cited with 68.2%, and 36.4% in the last seven days. When asked if pain prevented them from carrying out normal activities, such as work, housework or hobbies, in the last 12 months, the neck region had 27.3%.

Table 2 shows the partial results of the associations, through statistical analysis, using the chi-square test, between musculoskeletal symptoms and the gender variable reported by the nurses interviewed, highlighting the regions that presented statistically significant differences.

Observing Table 2, we can see the associations of musculoskeletal symptoms by region, in which we can see a significant difference (p=0.047<0.05) of the musculoskeletal symptom of the neck region in relation to the gender variable between the female and male groups in the period of the last 12 months. Likewise, we observed a significant difference (p=0.047<0.05) in the impediment of performing common daily activities due to pain located in the elbow region. Regarding the other musculoskeletal symptoms associated with the gender variable, there was no significant difference.

In Table 3, we have descriptive data regarding the WHOQOL-BREF questionnaire domains, which describes nurses’ understanding in relation to each domain, calculating mean scores, standard deviation (SD), 95% Confidence Interval (CI), maximum and minimum values and the scores of the domains that have a similarity ranging from 55.74 (physical) to 65.34 (general QoL).

**Table 3:** Descriptive data of the quality of life domains answered by nurses through the WHOQOL-BREF questionnaire

Domains	Minimum	Maximum	Mean ± SD	95% CI
General quality of life	12.50	100.00	65.34 ± 17.79	60.97-69.71
Physical quality of life	39.29	71.43	55.74 ± 8.12	53.74 - 57.73
Psychological quality of life	41.67	87.50	60.67 ± 9.86	58.24 - 63.09
Social quality of life	8.33	100.00	63.13 ± 17.54	58.82 - 67.44
Environment quality of life	40.63	84.38	63.78 ± 9.35	61.48 - 66.08

*SD - standard deviation of the domains; CI - Confidence Interval.*

The WHOQOL-BREF questionnaire adopts a comprehensive view of QoL in multiple domains, such as physical, psychological, social, environment and general QoL, enabling a broad understanding of what is understood by respondents’ satisfaction, comfort and well-being. When comparing these domains to independent variables, as well as to some of the musculoskeletal symptoms felt by nurses, they are described in Table 4.

**Table 4:** Quality of life domains associated with independent variables of nurses from the Francisco Beltrão Health Region, Paraná, Brazil

Variables	Domains				
	Physical	Psychological	Social	Environment	General quality of life
Sex	0.321	0.073	0.290	0.053	0.593
Being married	0.798	0.394	0.754	0.222	0.630
Having children	0.925	0.403	0.276	0.944	0.723
Having another relationship	0.819	0.094	0.235	0.036^*^	0.457
Physical activity	0.128	0.990	0.402	0.290	0.836
Use of psychotropic drugs	0.760	0.453	0.029^*^	0.061	0.645
Use of medications after the start of the COVID-19 pandemic	0.659	0.010^*^	0.008^*^	0.035^*^	0.016^*^
Increase in medication dose after the start of the COVID-19 pandemic	0.039^*^	0.096	0.010^*^	0.025^*^	0.185
Low back pain	0.164	0.025^*^	0.001^*^	0.034^*^	0.025^*^
Neck pain	0.818	0.039^*^	0.072	0.152	0.029^*^
Pain in the thoracic region	0.949	0.009^*^	0.000^*^	0.051	0.308
Shoulder pain	0.336	0.270	0.018^*^	0.266	0.064

* *Significance level (p<0.05).*

Thus, we see in Table 4 the results of research analyses in which non-parametric tests were adopted, such as Kruskal-Wallis test and Mann-Whitney U test, with correction, in which the domains were compared with sample variables.

Thus, gender, being married, having children and practicing physical activity do not influence any of the domains addressed. The variable that questions whether the professional has another employment relationship, associated with the domains, resulted in a significance with the environment domain (p = 0.036<0.05), which is characterized by physical protection, financial resources and transportation, and the physical environment itself, characterized by pollution, noise, climate and traffic.

When questioning the use of psychotropic medications, it was found that their use was significant in the social domain (p = 0.029 < 0.05). The start of the use of these medications after the beginning of the COVID-19 pandemic was statistically significant in the psychological (p = 0.010 < 0.05), social (p = 0.008 < 0.05), environment (p = 0.03 < 0.05) and general QoL (p = 0.016 < 0.05) domains. Questioning the increase in the dose of medication after the beginning of the pandemic influenced the physical (p = 0.039 < 0.05), social (p = 0.010 < 0.05) and environment (p = 0.025 < 0.05) domains.

Assessing the variable of low back pain, which was the most cited by participants, it is clear that this pain interferes with the psychological (p = 0.025<0.05), social (p = 0.001<0.05), environment (p = 0.034<0.05) and general QoL (p = 0.025<0.05) domains. Neck pain influences the psychological (p = 0.039<0.05) and general QoL (p = 0.029<0.05) domains. Pain in the chest region affects the psychological (p = 0.009<0.05) and social (p = 0<0.05) domains, and shoulder pain has significance with the social domain (p = 0.018<0.05).

## DISCUSSION

QoL is influenced by several factors, including economic status, physical and mental well-being, good coexistence in society and in the work environment, and family life^(18)^. One of the factors for the decline in QoL is professional stress. Nurses face situations that may involve contact with death, conflict with colleagues and management, affecting their health and QoL^(19)^.

Over time, scientific discoveries were documented, and care began to be provided based on knowledge. This was possible due to a woman known as Florence Nightingale, who was a precursor to this new form of care, thus giving rise to nursing based on scientific knowledge^(20)^.

Based on this, this profession has an epidemiological profile that repeats itself over the years, and is evidenced in studies, being mostly composed of women with a steady partner or married^(21-24)^. Due to cultural divisions since ancient times regarding professions, nursing was seen as fragile and subordinate, and even having its thinking directed towards charity, with the feminization of this profession^(25-27)^.

In this study, we sought to identify whether these professionals use psychotropic medications, and a percentage of them stated that they do, a finding that supports a study conducted with nurses in Rio de Janeiro, in which 36.8% reported using psychotropic drugs in the last month. The unbridled use of these medications, without specialized medical supervision, consists of the provision and access to them^(28)^. In a survey conducted with professionals from all over Brazil, it was found that 25.90% started using sleeping medications due to increased workload and psychological overload^(29)^. However, psychotherapy, which can be used as a treatment and prevention of mental illnesses, such as anxiety, was reported by a small portion (15.39%) of this population. However, it is worth noting that psychotherapy is one of the best forms of treatment for stress, as it seeks strategies for professionals to face problems in a way that their mental health is not so impacted^(30)^.

With the increase in the workload for healthcare professionals, specifically nurses, there is a lack of concern for their own health, in which they end up dedicating their lives to caring for other people and forgetting about their own needs. As a result, there is an increase in muscle pain^(22)^. These professionals continually prioritize their patients and, on certain occasions, do not use adequate ergonomics to provide care, thus causing injuries and pain. These are the professionals who are always in contact with the population, providing care, and being the front line in patient care^(31)^.

Muscle pain is present in the entire population, regardless of its intensity and frequency. However, a greater increase is observed among healthcare professionals. This study indicates that lower back pain was the most cited in the last 12 months and also in the last seven days. This data supports a study carried out in Istanbul, where pain in the lower back region was the most cited by nurses who reported pain, with 90.4%^(32)^. However, this study differs from a study carried out with Iranian nurses, in which lower back pain was in second place as the most manifested pain, with the most cited pain being in the ankles/feet. However, in the assessment of the last seven days, this region was the most cited by the same nurses^(33)^. In a study carried out by Lopes *et al*.^(34)^ with 451 workers from a federal public institution in the southern region of the country, the estimated prevalence of musculoskeletal symptoms in the last 12 months of the research was 90%. However, there was a prevalence of females and physical inactivity, and workers classified as having a low work capacity index had more symptoms. It is worth noting that technical education acted as a protective factor, reducing the mean of muscular symptoms by 36.46%^(34)^.

The discomfort reported by the population studied, in the lower back region associated with the work environment, can be explained by the fact of remaining in the same position for a considerable time, and ergonomics itself, which is not practiced correctly, associated with tension, the weight of inappropriate objects, inadequate lifting of patients and incorrect postures, contributes to developing this pain^(10,24)^.

The second most cited segmentation for causing pain was the neck region. This data supports a study carried out with professionals living in the state of São Paulo and who provide their services in PHC. These muscular symptoms presented by professionals are due to long working hours, shortened breaks, repetitive movements and inadequate posture for performing their functions^(35)^.

However, studies indicate that increased muscle pain causes a decline in the QoL of this individual, specifically the physical domain, which obtained the lowest mean in the present study, a finding similar to a study conducted with 668 nurses in Poland, where the mean for the physical domain was 62.13^(36)^. A study conducted in Paraná with professionals working in primary care shows that the physical domain was the one that obtained the highest score in the classification, called regular^(37)^.

A good QoL results in greater personal and work productivity, generating satisfaction for oneself and others, and helping to improve the quality of care provided^(38)^. A decline in QoL can be caused by several factors, such as professional devaluation, high workload, insufficient number of professionals, low salaries, the workplace itself and little social support^(39,40)^.

There was no significant relationship between QoL and gender, a finding that supports the study by Orszulak (2022), since, in the individual assessment of the domains, the studies agreed that the physical domain was the one that obtained the lowest mean^(18)^. However, in the study by Caliari (2022), carried out with nursing professionals from all Brazilian regions, where the physical domain was the one that obtained the highest mean (59.77), in more than one employment relationship, the QoL domains had a decrease, except for the psychological one^(29)^, in which the present study showed that more than one relationship affects environment QoL.

Healthcare professionals are still suffering the consequences of the COVID-19 pandemic, in which they have taken on more responsibilities and had to work longer hours to serve the entire population. However, a study conducted with these professionals shows that there was no significant difference in the QoL of professionals who worked on the front lines during COVID-19^(41)^. However, we need to consider that any change in routine and experience contributes to a natural imbalance in the lives of these professionals.

### Study limitations

It is extremely important to consider that the study had limitations. Since it was a regional study, covering all municipalities, there were differences in the number of professionals participating in each municipality. Moreover, it was a cross-sectional study, which made it impossible to establish cause-and-effect relationships between a condition and its risk factors or causes. Therefore, further studies are suggested to better understand the scenario.

### Contributions to nursing, health or public policy

The need to improve the working conditions of the professionals covered in this research is notable as well as their knowledge about these situations. It is understood that, at the moment when a person begins to analyze their actions and situations, they may realize their own mistake, thus proposing to change. A good QoL benefits not only professionals, but mainly the population.

## CONCLUSIONS

It is concluded that professionals, based on the knowledge they have about their QoL, believe that it is superior to reality and that their QoL cannot be considered good. Furthermore, muscle pain is present especially in the lower back and neck region in most nurses. This pain ends up influencing several domains of QoL as well as the use of psychotropic medication.

These professionals need to seek measures to improve these rates. It is important to emphasize the need for public policies aimed at improving the health team’s QoL as well as maintaining staffing levels, avoiding team overload.
