# Supplementary material for: Quality of life and musculoskeletal symptoms of nurses in Primary Care in southern Brazil
Source: Rev Bras Enferm. 2025 Mar 14;78(1):e20230342. doi: 10.1590/0034-7167-2023-0342 (PMC11913047; doi:10.1590/0034-7167-2023-0342)
Supplement: Translation

# Translation: Qualidade de vida e sintomas osteomusculares de enfermeiros na Atenção Primária do sul do Brasil

**DOI:** 10.1590/0034-7167-2023-0342pt

**Authors:** Jadieli Simoni Roll-Koch, Lirane Elize Defante Ferreto, Adilson Carlos da Rocha, Rosebel Trindade Cunha Prates, Dalila Moter Benvegnú, Gisele Arruda, Ana Paula Vieira, Franciele Aní Caovilla Follador

**Affiliations:** I Universidade Estadual do Oeste do Paraná. Francisco Beltrão, Paraná, Brasil; II Universidade Federal da Fronteira Sul. Realeza, Paraná, Brasil

**Keywords:** Qualidade de Vida, Dor Musculoesquelética, Enfermeiras e Enfermeiros, Atenção Primária à, Saúde, Saúde do Trabalhador

## Abstract

**Objetivos:**

avaliar o conhecimento sobre qualidade de vida e sintomas osteomusculares de enfermeiros atuantes na Atenção Primária à Saúde da Regional de Francisco Beltrão, Paraná.

**Métodos:**

trata-se de pesquisa exploratória, quantitativa, transversal, realizada com enfermeiros, utilizando questionário sociodemográfico, WHOQOL-BREF e Questionário Nórdico de Sintomas Osteomusculares. A análise estatística utilizouo programa estatístico *Statistical Package for the Social Sciences,* aplicando teste qui-quadrado, teste Kruskal-Wallis e teste U de Mann-Whitney.

**Resultados:**

a amostra constituiu-se de 66 enfermeiros, sendo a maioria mulheres (86,4%), casadas (71,2%) e idade média de 37,23 anos. Quando avaliada a qualidade de vida, o domínio físico obteve a menor média (55,74), seguido do domínio psicológico (60,67). Em relação aos sintomas osteomusculares, a dor na região lombar foi a mais citada (75,80%).

**Conclusões:**

o conhecimento que os enfermeiros têm sobre a QV mostra que esta é influenciada pelas dores musculares, por ter outro vínculo empregatício e uso de psicotrópicos.

## INTRODUÇÃO

A Atenção Primária à Saúde (APS) é responsável pelo atendimento do maior contingente populacional, com diferentes demandas de saúde. Estes profissionais atuam na prevenção, promoção e recuperação da saúde da população, muitas vezes com poucos recursos e altas demandas. Entre os profissionais da equipe que compõem a linha de atenção básica, encontra-se o enfermeiro. Geralmente, esse profissional é responsável pela gestão, organização e atendimento da população, ou seja, inúmeras atividades para executar no horário de trabalho^(1)^.

A APS é de essencial importância, pois a mesma objetiva o atendimento integral do indivíduo, sendo a porta de entrada do mesmo para os serviços de saúde^(2)^. Além deste atendimento integral, constitui-se por estratégias para a melhor prestação deste serviço, como a Estratégia Saúde da Família (ESF) e a Unidade de Saúde da Família (USF)^(3)^.

O próprio Ministério da Saúde (MS) reforça a importância do cuidado prestado por equipes multiprofissionais e, nestas, fica evidenciada a importância do papel exercido pelo enfermeiro como gestor e assistencialista nas ações de prevenção e promoção da saúde. Entretanto, por muitas vezes, a dedicação ao melhoramento da população assistida não é visualizada em si mesmo^(1)^.No momento em que o cuidado pessoal não assume uma prioridade em sua vida, tem-se um declínio da qualidade de vida (QV) desse profissional^(4)^.

A QV, segundo a Organização Mundial da Saúde (OMS), é “[...] a percepção do indivíduo de sua posição na vida no contexto da cultura e sistema de valores nos quais ele vive e em relação aos seus objetivos, expectativas, padrões e preocupações”^(5)^. Sendo assim, a mesma sofre influência de fatores externos e internos, como dor e desconfortos, relações sociais, o curso do pensamento, a segurança física e o lazer^(6)^ .

Os enfermeiros têm o conhecimento prévio sobre QV, entanto, em sua maioria, estão insatisfeitos e relatam ter dificuldade de manter um equilíbrio entre a vida pessoal e a profissional^(1)^. Estes ainda sabem da importância de ter um tempo destinado às atividades de lazer, que não esteja vinculado ao ambiente e processo de trabalho, entretanto a falta de tempo não favorece a realização das mesmas, interferindo na vida pessoal e até mesmo no cuidado prestado ao paciente^(4)^. Já na atualidade, podemos citar que a pandemia de COVID-19 influenciou a falta de convívio social e a realização de atividades não domiciliares. Com a propagação descontrolada do vírus e a falta de cura e de imunobiológico para prevenção, toda população experienciou a quarentena^(6)^.

Com a exposição continuada a estressores e longas jornadas de trabalho, consequentemente, esses profissionais começaram a apresentar dores musculares causadas por tensão, postura inadequada e a própria repetitividade de uma ação sem ergonomia adequada^(7)^. A doença osteomuscular relacionada ao trabalho (DORT) é a sequência de lesões pelo esforço repetitivo (LER) musculares não tratadas e não prevenidas. São consideradas as maiores responsáveis pelo absenteísmo e presenteísmo profissional, além de ser resultantes de escassez profissional e ambientes não adaptados ergonomicamente^(8)^. Ainda estudo realizado no Vietnã afirma que as profissionais femininas estão mais predispostas ao desenvolvimento de LER e DORT, devido à dupla jornada, como os afazeres domésticos^(9)^. Consequentemente, essa sintomatologia resulta em falhas na atenção ao paciente, automedicação e também diminuição da QV^(10)^. Com esta problemática, em que o enfermeiro desempenha funções diversificadas em seu ambiente laboral, bem como a jornada de trabalho, faz-se necessário compreender como encontra-se a QV deste profissional, assim como os sintomas decorrentes do trabalho e a periodicidade de dor, buscando conhecimento para que a classe e o sistema busquem melhorias para minimizar tais ocorrências.

## OBJETIVOS

Avaliar o conhecimento sobre a QV e sintomas osteomusculares de enfermeiros atuantes na APS da Regional de Francisco Beltrão, Paraná.

## MÉTODOS

### Aspectos éticos

O estudo possui aprovação pelo Comitê de Ética em Pesquisa (CEP) da Universidade Estadual do Oeste do Paraná (UNIOESTE), sendo que os dados só foram coletados após assinatura do Termo de Consentimento Livre e Esclarecido.

### Desenho, local do estudo e período

Trata-se de pesquisa exploratória, de caráter quantitativo e transversal, realizada com profissionais enfermeiros atuantes na APS da Regional de Saúde de Francisco Beltrão, no Paraná. Esta regional de saúde contempla um total de 27 municípios, sendo integrante da macrorregional Oeste do Paraná^(11)^. Nesta regional de saúde, a APS é constituída por 213 enfermeiros, conforme dados obtidos no Cadastro Nacional de Estabelecimentos de Saúde (CNES) em março de 2021^(12)^. A coleta de dados foi realizada entre maio de 2021 e março de 2022, período este da pandemia de COVID-19. Além disso, seguiram-se as orientações da *STrengthening the Reporting of OBservational studies in Epidemiology*(STROBE) para publicação de estudos observacionais^(13)^.

### Amostra, critérios de inclusão e exclusão

A amostra se constituiu por conveniência/aceitação. Inicialmente, a forma de coleta de dados apresentou-se de forma *online*, auxiliada pela ferramenta *Google Forms^®^.* O *link* contendo os questionários foi encaminhado para todos os enfermeiros por intermédio de e-mail e/ou *WhatsApp^®^*. Após a baixa adesão, foi realizada a coleta, de forma presencial, em todos os municípios. Sendo assim, a amostra foi composta por 66 enfermeiros atuantes na APS.

### Protocolo do estudo

Para este estudo, foram usados como instrumentos para coleta de dados o questionário sociodemográfico e epidemiológico, os quais abordaram dados como, idade, sexo, estado marital, tempo de serviço, renda familiar mensal, se possui pós-graduação, realização de atividade física, religião, outros vínculos empregatícios, quantitativo de horas semanais trabalhadas, se exerce a sua profissão no período noturno, doenças de base, medicamentos utilizados para saúde mental e se iniciou o uso dessas medicações após o início da pandemia.

No intuito de avaliar a QV do profissional enfermeiro, foi utilizado o questionário WHOQOL-BREF, sendo uma abreviação do WHOQOL-100^(14)^. No ano de 2000, este questionário foi traduzido, aplicado na versão português e validado pela literatura brasileira^(15)^. O mesmo é composto por 26 perguntas, sendo que dois questionamentos se referem à QV no geral e ao estado de saúde, e as demais 24 questões avaliam quatro domínios separadamente, sendo: físico; psicológico; relações sociais; e meio-ambiente^(14)^.

Para avaliação dos sintomas osteomusculares, foi utilizado o Questionário Nórdico de Sintomas Musculoesqueléticos (QNSM). Neste estudo, foi utilizada a versão traduzida e validada para a língua portuguesa por Mesquita (2010)^(16)^. Esse questionário avalia se o pesquisado apresentou sintomatologia de dor, desconforto ou dormência nos seus devidos segmentos corporais nos últimos 12 meses e nos sete dias anteriores ao preenchimento do formulário. Ainda, questiona-se se esse funcionário teve que se afastar ou evitar as suas atividades normais, motivado pelos problemas osteomusculares nos segmentos^(17)^.

A literatura já demonstrou que os questionários utilizados, embora na versão portuguesa, apresentaram semelhança nos resultados das características psicométricas^(15)^. Nesse sentido, justifica-se o uso em função da disponibilidade do instrumento e da semelhança entre a semântica das línguas, não sendo observado, no decorrer da pesquisa, nenhum relato dos participantes sobre nenhum problema de interpretação em função do uso. Nesse sentido, não se observou nenhum prejuízo no uso da versão portuguesa.

### Análise dos resultados e estatística

Os questionários adotados e aplicados aos enfermeiros foram codificados com o auxílio do *software Microsoft Excel* ^®^, e, para as análises estatísticas e associações, utilizou-se o programa estatístico *Statistical Package for the Social Sciences* (SPSS), versão 23, no qual foram aplicados testes não paramétricos, como o teste qui-quadrado, teste Kruskal-Wallis e teste U de Mann-Whitney, com correção para amostra.

## RESULTADOS

A amostra foi composta por 66 profissionais enfermeiros, a maioria (86,4%) do gênero feminino, com idade média de 37,23, sendo a mínima 23 e máxima 56 anos. Destes, 71,2% eram casados, de cor branca (94,4%), de religião católica (81,1%), com um filho (37,9%) e renda familiar de mais de quatro salários mínimos (60,6%).

Quanto ao tempo de formação desses profissionais, a maioria era formada há mais de dez anos (59,1%) e com pós-graduação (84,8%). Evidenciou-se que 53% atuavam na área há mais de dez anos; poucos deles possuíam outro vínculo empregatício (12,1%); e atuavam em período noturno (12,1%). Além disso, a maioria (86,4%) trabalhava de 30 a 44 horas semanais.

A atividade de lazer periódica foi relatada por 83,3% dos participantes. Já a prática regular de atividade física foi citada por somente 42,4% desses. Quando questionados sobre o uso de álcool, 57,6% informaram realizar o uso, no mínimo, socialmente, e 93,9% relataram não serem tabagistas. Uma parte desses profissionais (24,2%) afirmou ter doenças de base, sendo citadas a hipertensão arterial sistêmica (HAS), diabetes mellitus (DM), asma, cardiopatia, hipotireoidismo, psoríase, doença pulmonar obstrutiva crônica (DPOC), obesidade, tremor essencial de ação, LER e DORT.

A presença de doença mental foi informada por 22,7%, no entanto, de todos os entrevistados, 28,8% relataram o uso de psicotrópicos, e apenas 15,2% afirmaram a realização de psicoterapia. Ao questionamento de início de uso de medicações psicotrópicas após o começo da pandemia, apenas 7,6% afirmaram, e 15,2% já usavam a medicação e aumentaram a dose durante a pandemia. Ao serem questionados se houve mudança na QV com a vivência da pandemia, 75,8 referiram que sim, sendo a mesma para pior.

As dores musculares são desconfortos que podem acometer toda a população. Para identificarmos a prevalência de sintomas osteomusculares dos enfermeiros, adotou-se o QNSM,no qual obtiveram-se os resultados descritos nas Tabelas 1 e 2.

**Tabela 1:** Sintomas osteomusculares descrito pelos enfermeiros da Regional de Saúde de Francisco Beltrão, Paraná, Brasil

Local/região da dor	Dor últimos 12 meses		Impedimento na realização das atividades nos últimos 12 meses		Dor nos últimos sete dias	
	n	%	n	%	n	%
Pescoço	45	68,2	18	27,3	24	36,4
Ombros ambos	28	42,4	5	7,6	9	13,6
Ombro direito	8	12,1	7	10,6	6	6,1
Cotovelo ambos	2	3,0	2	3,0	2	3,0
Cotovelo direito	7	10,6	3	4,5	2	3,0
Cotovelo esquerdo	1	1,5	1	1,5	-	-
Punhos e mãos ambos	8	12,1	2	3,0	1	1,5
Punho e mão direito	9	13,6	5	7,6	3	4,5
Punho e mão esquerda	3	4,5	1	1,5	1	1,5
Região torácica	20	30,3	7	10,6	10	15,2
Região lombar	50	75,8	22	33,3	26	39,4
Ancas e coxas	8	12,1	3	4,5	5	7,6
Joelhos	20	30,3	7	10,6	10	15,2
Tornozelo e pés	11	16,7	2	3,0	5	7,6

**Tabela 2:** Associação de sintomas osteomusculares por região anatômica em relação ao sexo dos enfermeiros da Regional de Saúde de Francisco Beltrão, Paraná, Brasil

			Sexo				Valor de *p*
			Feminino		Masculino		
			n	%	n	%	
Pescoço	12m		42	66,64	3	4,54	0,047^*^
	7d		22	33,33	2	3,03	0,476
	Impedimento		17	25,76	1	1,52	0,317
Ombros	12m		32	48,48	4	6,06	0,954
	7d		12	18,18	2	3,03	0,667
	Impedimento		10	15,15	2	3,03	0,284
Cotovelos	12m		8	12,12	2	3,03	0,054
	7d		4	6,06	0	0,00	0,746
	Impedimento		5	7,58	1	1,52	0,047^*^
Punhos e mãos	12m		18	27,27	2	3,03	0,928
	7d		4	6,06	1	1,52	0,667
	Impedimento		8	12,12	0	0,00	0,740
Torácica	12m		18	27,27	2	3,03	0,728
	7d		10	15,15	0	0,00	0,202
	Impedimento		6	9,09	1	1,52	0,853
Lombar	12m		44	66,67	6	9,09	0,957
	7d		24	36,36	2	3,03	0,374
	Impedimento		21	31,82	1	1,52	0,182
Quadris e coxas	12m		8	12,12	0	0,00	0,262
	7d		5	7,58	0	0,00	0,388
	Impedimento		3	4,54	0	0,00	0,510
Joelhos	12m		16	24,24	4	6,06	0,823
	7d		9	13,63	1	1,52	0,196
	Impedimento		4	6,06	3	4,54	0,080
Tornozelos e pés	12m		9	13,64	2	3,03	0,500
	7d		4	6,06	1	1,52	0,574
	Impedimento		1	1,52	1	1,52	0,096

* *Diferença significativa em nível de 5% (p<0,05).*

Os dados apresentados na Tabela 1 são referentes aos sintomas osteomusculares relatados pelos enfermeiros. Ao questionamento se tiveram algum problema tal como dor, desconforto ou dormência nos últimos 12 meses, a região lombar foi a mais citada (75,80%); considerando os últimos sete dias, a mesma foi citada em 39,4%. Já ao questionar se a dor impediu de realizar atividades normais, como trabalho, serviço doméstico ou passatempos, nos últimos 12 meses, esta foi citada em 33,30%. Em seguida, em relação à posição como dor, desconforto ou dormência, nos últimos 12 meses, a região do pescoço foi citada com 68,2%, e 36,4% nos últimos sete dias. Quando questionados se a dor impediu de realizar atividades normais, como trabalho, serviço doméstico ou passatempos, nos últimos 12 meses, a região do pescoço teve 27,3%.

Na Tabela 2, encontram-se os resultados parciais das associações, mediante análise estatística, pelo teste qui-quadrado, entre sintomas osteomusculares e a variável sexo referidas aos enfermeiros entrevistados, destacando-se as regiões que apresentaram diferenças estaticamente significativas.

Observando a Tabela 2, percebem-se as associações dos sintomas osteomusculares por região, na qual pode-se visualizar uma diferença significativa (*p* = 0,047 <0,05) do sintoma osteomuscular da região do pescoço em relação à variável sexo entre o grupo feminino e masculino no período dos últimos 12meses.Da mesma forma, observou-se uma diferença significativa *(p* = 0,047 <0,05) no impedimentode realização de atividades diárias comum pela dor localizada na região do cotovelo. Já em relação aos outros sintomas osteomusculares associados à variável sexo, não houve nenhuma diferença significativa.

Na Tabela 3, temos dados descritivos referentes aos domínios do questionário WHOQOL-BREF, que descreve o entendimento dos enfermeiros em relação a cada domínio, sendo calculados os escores médios, desvio padrão (DP), Intervalo de Confiança de 95% (IC), valores máximos, mínimos e os escores dos domínios que têm uma similaridade variando de 55,74 (físico) a 65,34 (QV geral).

**Tabela 3:** Dados descritivos dos domínios da qualidade de vida respondidas pelos enfermeiros através do questionário WHOQOL-BREF

Domínios	Mínimo	Máximo	Média ± DP	IC95%
Qualidade de vida geral	12,50	100,00	65,34 ± 17,79	60,97-69,71
Qualidade de vida físico	39,29	71,43	55,74 ± 8,12	53,74 - 57,73
Qualidade de vidapsicológico	41,67	87,50	60,67 ± 9,86	58,24 - 63,09
Qualidade de vida social	8,33	100,00	63,13 ± 17,54	58,82 - 67,44
Qualidade de vida ambiental	40,63	84,38	63,78 ± 9,35	61,48 - 66,08

*DP - desvio padrão dos domínios; IC -Intervalo de Confiança.*

O questionário WHOQOL-BREF adota uma visão abrangente da QV em múltiplos domínios, como físico, psicológico, social, ambiental e QV geral, viabilizando uma compreensão ampla do que se entende por satisfação, conforto e bem-estar dos pesquisados. Ao compararmos esses domínios às variáveis independentes, bem como a alguns dos sintomas osteomusculares sentidos pelos enfermeiros, os mesmos estão descritos na Tabela 4.

**Tabela 4:** Domínios da qualidade de vida associados às variáveis independentes de enfermeiros da Regional de Saúde de Francisco Beltrão, Paraná, Brasil

Variáveis	Domínios				
	Físico	Psicológico	Social	Ambiental	Qualidade de vida geral
Sexo	0,321	0,073	0,290	0,053	0,593
Ser casado	0,798	0,394	0,754	0,222	0,630
Ter filhos	0,925	0,403	0,276	0,944	0,723
Ter outro vínculo	0,819	0,094	0,235	0,036^*^	0,457
Prática de atividade física	0,128	0,990	0,402	0,290	0,836
Uso de psicotrópicos	0,760	0,453	0,029^*^	0,061	0,645
Uso de medicações após o início da pandemia de COVID-19	0,659	0,010^*^	0,008^*^	0,035^*^	0,016^*^
Aumento de dose de medicação após o início da pandemia de COVID-19	0,039^*^	0,096	0,010^*^	0,025^*^	0,185
Dor lombar	0,164	0,025^*^	0,001^*^	0,034^*^	0,025^*^
Dor no pescoço	0,818	0,039^*^	0,072	0,152	0,029^*^
Dor em região torácica	0,949	0,009^*^	0,000^*^	0,051	0,308
Dor no ombro	0,336	0,270	0,018^*^	0,266	0,064

* *Nível de significância (p<0,05).*

Desta forma, vemos na Tabela 4 os resultados das análises de pesquisa em que foram adotados testes não paramétricos, como teste Kruskal-Wallis e o teste U de Mann-Whitney, com correção, em que os domínios foram comparados com as variáveis da amostra.

Assim, sexo, ser casado, possuir filhos e prática de atividade física não influenciam nenhum dos domínios abordados. A variável que questiona se o profissional possui outro vínculo empregatício, associado aos domínios, resultou em uma significância com o domínio ambiental (*p* = 0,036 <0,05), que é caracterizado pela proteção física, recursos financeiros e transporte, e o próprio ambiente físico, caracterizado pela poluição, ruídos, clima e trânsito.

Ao questionar o uso de medicações psicotrópicas, verificou-se que o uso desses possui significância com o domínio social (*p* = 0,029 <0,05). O início do uso dessas medicações após o começo da pandemia de COVID-19teve estatisticamente significância nos domínios psicológico (*p* = 0,010 <0,05) , social (*p* = 0,008 <0,05), ambiental (*p* = 0,03 <0,05) e QV geral (*p* = 0,016 <0,05). Questionar o aumento da dose da medicação, após o início da pandemia, influenciou os domínios físico (*p* = 0,039 <0,05), social (*p* = 0,010 <0,05) e ambiental (*p* = 0,025 <0,05).

Avaliando a variável de dor lombar, que foi a mais citada pelos participantes, percebe-se que essa dor interfere nos domínios psicológico (*p* = 0,025 <0,05), social (*p* = 0,001 <0,05), ambiental (*p* = 0,034 <0,05) e QV geral (*p* = 0,025 <0,05). Já a dor no pescoço influencia os domínios psicológico (*p* = 0,039 <0,05) e QV geral (*p* = 0,029 <0,05). A dor na região de tórax afeta os domínios psicológico (*p* = 0,009 <0,05) e social (*p* = 0 <0,05), e a dor em ombros possui significância com o domínio social (*p* = 0,018 <0,05).

## DISCUSSÃO

A QV tem interferência de vários fatores, incluindo a condição econômica, o bem-estar físico e mental, a boa convivência em sociedade e no próprio ambiente de trabalho, e a vida familiar^(18)^. Um dos fatores para o declínio da QV é o estresse profissional. O enfermeiro enfrenta situações que podem ter o contato com a morte, conflito com colegas e chefia, afetando a sua saúde e QV^(19)^.

Com o passar do tempo, as descobertas científicas foram documentadas, e então o cuidado passou a ser prestado embasado em saberes. Isto foi possível devido a uma mulher conhecida como Florence Nightingale, que é precursora dessa nova forma de cuidar, originando, assim, a enfermagem baseada em saberes científicos^(20)^.

Com base nisto, essa profissão possui um perfil epidemiológico que se repete ao decorrer de anos, e é evidenciado nos estudos, sendo em sua maioria composto por mulheres e com companheiro fixo ou casadas^(21-24)^. Devido às divisões culturais desde a antiguidade referentes às profissões, a enfermagem foi vista como frágil e subordinada, e até mesmo tendo o pensamento direcionado à caridade, coma feminilização dessa profissão^(25-27)^.

Neste estudo, buscou-se identificar se esses profissionais fazem uso de medicações psicotrópicas, sendo que uma porcentagem desses afirmou o uso, dado este que corrobora estudo realizado com enfermeiros do Rio de Janeiro, em que 36,8% relataram fazer uso de psicotrópicos no último mês. O uso desenfreado dessas medicações, sem acompanhamento médico especializado, consiste na oferta e acesso aos mesmos^(28)^. Em pesquisa realizada com profissionais de toda Brasil, constatou-se que 25,90% iniciaram uso de medicações para dormir motivados pelo aumento do trabalho e sobrecarga psicológica^(29)^. No entanto, a psicoterapia, que pode ser usada como tratamento e prevenção de doenças mentais, como a ansiedade, foi relatada por uma pequena parcela (15,39%) dessa população. Entanto, vale ressaltar que a psicoterapia é uma das melhores formas de tratamento para o estresse, pois busca estratégias para o profissional enfrentar os problemas de forma que a sua saúde mental não seja tão impactada^(30)^.

Com o aumento da carga de trabalho para os profissionais de saúde, especificamente enfermeiros, vê-se o descaso com a sua própria saúde, em que os mesmos acabam dedicando a vida a cuidar de outras pessoas e esquecendo de suas próprias necessidades. Com isso, tem-se o aumento de dores musculares^(22)^. Continuamente, esse profissional tem o seu paciente como prioridade, e, em certas ocasiões, não usa a ergonomia adequada para prestar o atendimento, gerando, assim, lesões e dores. Este é o profissional que está sempre em contato com a população, prestando os cuidados, sendo a linha de frente nos atendimentos aos pacientes^(31)^.

As dores musculares estão presentes em toda população, independentemente da intensidade e frequência dela, no entanto, em profissionais de saúde, percebe-se um maior aumento. O presente estudo aponta que as dores lombares foram as mais citadas nos últimos 12 meses e também nos últimos sete dias. Este dado corrobora estudo realizado em Instambul, onde a algia em região lombar foi a mais citada pelos enfermeiros que relataram dor, com 90,4%^(32)^. Entretanto, este estudo diverge de estudo realizado com enfermeiras iranianas, em quea dor lombar estava em segunda colocação como a dor que mais se manifestava, sendo a dor mais citada a de tornozelos/pés. Contudo, na avaliação dos últimos sete dias, essa região foi a mais citada pelas mesmas^(33)^. Já no estudo realizado por Lopes *et al*.^(34)^ com 451 trabalhadores de instituição pública federal na região Sul do país, a prevalência estimada de sintomas osteomusculares nos últimos 12 meses da realização da pesquisa foi de 90%. Entanto, houve prevalência do sexo feminino e inatividade física, e os trabalhadores classificados com índice de capacidade de trabalho baixo tiveram mais sintomas. Vale ressaltar que escolaridade de ensino técnico atuou como fator de proteção, reduzindo a média em 36,46% de sintomas musculares^(34)^.

O desconforto citado pela população estudada, na região inferior das costas associado ao meio laboral, pode ser explicado pelo fato de permanecer por um tempo considerável na mesma posição, e a própria ergonomia, que é não praticada corretamente, associada à tensão, ao peso de objetos desapropriados, elevação inadequadas de pacientes e posturas incorretas, auxilia no desenvolvimento dessa algia^(10,24)^.

A segunda segmentação mais citada por causar dor foi a região de pescoço. Este dado corrobora estudo realizado com profissionais residentes no estado de São Paulo e que prestam seus atendimentos em APS. Esses sintomas musculares apresentados pelos profissionais são derivado de longas jornadas de trabalho, pausas encurtadas, movimentos repetitivos e postura inadequada para a realização de suas funções^(35)^.

No entanto, estudos apontam que o aumento de dores musculares ocasiona o declínio na QV deste indivíduo, especificamente o domínio físico, que obteve a menor média no presente estudo, dado este semelhante a pesquisa realizada com 668 enfermeiros na Polônia, onde a média do domínio físico foi de 62,13^(36)^. Já estudo realizado no Paraná com os profissionais que atuam na atenção primária demonstra que o domínio físico foi o que obteve a maior pontuação na classificação, denominada regular^(37)^.

Uma boa QV resulta em maior produtividade pessoal e no trabalho, gerando satisfação própria e de terceiros, e auxiliando no melhoramento da qualidade do cuidado prestado^(38)^. O declínio da QV pode ser ocasionado por diversos fatores, como a desvalorização profissional, a elevada carga de trabalho, o número insuficiente de profissionais, os baixos salários, o próprio local de trabalho e pouco suporte social^(39,40)^.

Não houve relação significativa da QV em relação ao gênero, dado que corrobora o estudo de Orszulak (2022), uma vez que, na avaliação individual dos domínios, as pesquisas concordaram que o domínio físico foi o que obteve a menor média^(18)^. No entanto, no estudo de Caliari (2022), realizado com profissionais de enfermagem de todas as regiões brasileiras, onde o domínio físico foi o que obteve a maior média (59,77), em mais de um vínculo empregatício, os domínios de QV tiveram uma queda, exceto pelo psicológico^(29)^, em que o estudo presente apresentou que mais de um vínculo afeta a QV ambiental.

Os profissionais de saúde ainda sofrem as consequências deixadas pela pandemia de COVID-19, em que os mesmos assumiram mais responsabilidades e tiveram aumento nas jornadas de trabalho para atender a toda a população. Todavia, estudo realizado com esses profissionais demonstra que não houve uma diferença significativa na QV dos profissionais que atuaram em linha de frente na COVID-19^(41)^. Porém, precisamos considerar que qualquer mudança na rotina e na vivência contribui para um desequilíbrio natural na vida desse profissional.

### Limitações do estudo

É de suma importância considerar que o estudo teve limitações. Por se tratar de pesquisa de abrangência regional, tendo cobertura de todos os municípios, houve diferenças em quantitativo de profissionais participantes de cada município, além de ser estudo transversal, não sendo possível estabelecer relações de causa e efeito entre uma condição e seus fatores de risco ou causas. Desse modo, sugerem-se novos estudos para melhor compreensão do cenário.

### Contribuições para as áreas da enfermagem, saúde ou políticas públicas

É notável a necessidade de melhoramento das condições de trabalho dos profissionais abordados nesta pesquisa, bem como o conhecimento destes sobre essas situações. Entende-se que, no momento em que a pessoa inicia a análise de suas ações e situações, ela pode perceber o equívoco próprio, propondo-se, assim à mudança. Uma boa QV beneficia não somente os profissionais, mas principalmente a população.

## CONCLUSÕES

Conclui-se que os profissionais, com base no conhecimento que têm sobre sua QV, acreditam que ela é superior à realidade e que sua QV não pode ser considerada boa. Ainda, as dores musculares estão presentes especialmente na região lombar e pescoço em boa parte dos enfermeiros. Essas dores acabam influenciando vários domínios da QV, como também o uso de medicação psicotrópica.

É necessário que esses profissionais busquem medidas para o melhoramento desses índices. Ressalta-se a necessidade de políticas públicas voltadas ao melhoramento da QV da equipe de saúde, como também manter o dimensionamento de pessoal, evitando a sobrecarga da equipe.
